# Societal Burden and Correlates of Acute Gastroenteritis in Families with Preschool Children

**DOI:** 10.1038/srep22144

**Published:** 2016-02-26

**Authors:** Lapo Mughini-Gras, Roan Pijnacker, Moniek Heusinkveld, Remko Enserink, Rody Zuidema, Erwin Duizer, Titia Kortbeek, Wilfrid van Pelt

**Affiliations:** 1 National Institute for Public Health and the Environment (RIVM), Centre for Infectious Disease Control (CIb), PO Box 1, 3720 BA Bilthoven, the Netherlands; 2 Utrecht University, Department of Infectious Diseases and Immunology, Yalelaan 1, De Uithof, 3584 CL Utrecht, the Netherlands; 3 VU University Medical Center, Department of Medical Microbiology and Infection Control, Van der Boechorststraat 7, 1081 BT Amsterdam, the Netherlands

## Abstract

Gastrointestinal infection morbidity remains high amongst preschool children in developed countries. We investigated the societal burden (incidence, healthcare utilization, and productivity loss) and correlates of acute gastroenteritis (AGE) in families with preschoolers. Monthly for 25 months, 2000 families reported AGE symptoms and related care, productivity loss, and risk exposures for one preschooler and one parent. Amongst 8768 child-parent pairs enrolled, 7.3% parents and 17.4% children experienced AGE (0.95 episodes/parent-year and 2.25 episodes/child-year). Healthcare utilization was 18.3% (children) and 8.6% (parents), with 1.6% children hospitalized. Work absenteeism was 55.6% (median 1.5 days) and day-care absenteeism was 26.2% (median 1 day). Besides chronic enteropathies, antacid use, non-breastfeeding, and toddling age, risk factors for childhood AGE were having developmental disabilities, parental occupation in healthcare, multiple siblings, single-parent families, and ≤12-month day-care attendance. Risk factors for parental AGE were female gender, having multiple or developmentally-disabled day-care-attending children, antimicrobial use, and poor food-handling practices. Parents of AGE-affected children had a concurrent 4-fold increased AGE risk. We concluded that AGE-causing agents spread widely in families with preschool children, causing high healthcare-seeking behaviours and productivity losses. Modifiable risk factors provide targets for AGE-reducing initiatives. Children may acquire some immunity to AGE after one year of day-care attendance.

Acute gastroenteritis (AGE) is caused by a variety of infectious agents and some non-infectious conditions, often presenting with diarrhoea and/or vomiting that may impair daily functioning. AGE is usually self-limiting unless complicated by dehydration and extraintestinal manifestations. Although AGE mortality is low in developed countries and is decreasing globally^1^, its morbidity remains high, particularly amongst preschool children during wintertime^2^,^3^. A naïve adaptive immune system and imperfect hygiene behaviours make children prone to gastrointestinal infections. Moreover, children attending day-care centres (DCCs), where the intensity of contacts amongst peers is considerable and the exposure to circulating pathogens is high^4^, are at approximately twice the risk for infectious AGE as those home-cared^5^,^6^. Moreover, children may spread AGE-causing agents within the household, with child-to-parent transmission occurring approximately once every three AGE episodes in children^7^, and with young children having a 3- to 8-fold increased risk for secondary AGE than adults^8^.

Several studies on the burden of AGE have been published in Europe^2^,^3^,^5^,^9^,^10^,^11^,^12^,^13^,^14^,^15^,^16^,^17^,^18^, the Americas^8^,^12^,^19^,^20^,^21^, Asia and Oceania^12^,^22^,^23^,^24^,^25^. They focused either on the whole community^3^,^9^,^10^,^15^,^16^,^17^,^18^,^19^,^20^,^21^,^22^,^24^,^26^ or on specific age groups, such as (primarily preschool) children^2^,^5^,^6^,^7^,^14^,^27^,^28^, adults and elderly^11^,^23^. Amongst those studies from developed countries using a comparable case definition, the monthly community incidence of AGE was 2.6–11.1%, corresponding to 0.3–1.5 episodes/person-year. No study, however, focused specifically on the burden of AGE in families with preschool children and collected paired data for preschool children and their parents, though virtually all studies highlighted an increased risk for AGE in children^3^,^9^,^10^,^15^,^16^,^17^,^18^,^19^,^20^,^21^,^22^,^24^,^26^, especially those attending DCCs^5^,^14^,^25^,^27^, and in women^3^,^10^,^12^,^15^,^17^,^19^,^20^,^21^,^22^,^24^. It has been suggested that the still high morbidity of childhood AGE in developed countries may have a significant societal impact as a result of increased expenditure for medical care, alternative care (e.g. babysitting), and productivity losses due to worktime lost^5^,^7^,^14^,^29^, especially given the increasing number of dual-income and single-parent families.

As families with preschool children are likely to account for a substantial portion of the AGE community burden, characterizing infection risks within the household is relevant to the public health endeavour. However, only a few studies have looked at more risk factors for childhood AGE than only general demographic characteristics^8^,^14^,^28^, but they focused on DCC attendees^28^, or subsets of children with severe symptoms requiring medical care^8^,^14^, which may not be entirely representative of AGE in the general population.

We performed a nationwide survey of families with preschool children to determine the societal burden and correlates of AGE in these children and their parents, including whether parents of AGE-affected children had an increased AGE risk. Our goal was to provide an evidence base for how and to what extent AGE in developed countries poses a burden on the family, on the healthcare system, and on the society as a whole, identifying also potential risk factors.

## Methods

### Study Design

We performed a retrospective, monthly-repeated cross-sectional survey of families with <4-year-old children conducted in the Netherlands during October 2012–October 2014 (25 months), the so-called “Family & Health” survey. The focus on <4-year-old children is because at four years children become eligible to enrol in primary school in the Netherlands. Households were randomly selected from population registries of 335/415 municipalities covering 78% of the Dutch population (~16.8 million, ~5% aged <4 years).

**Figure 1:**
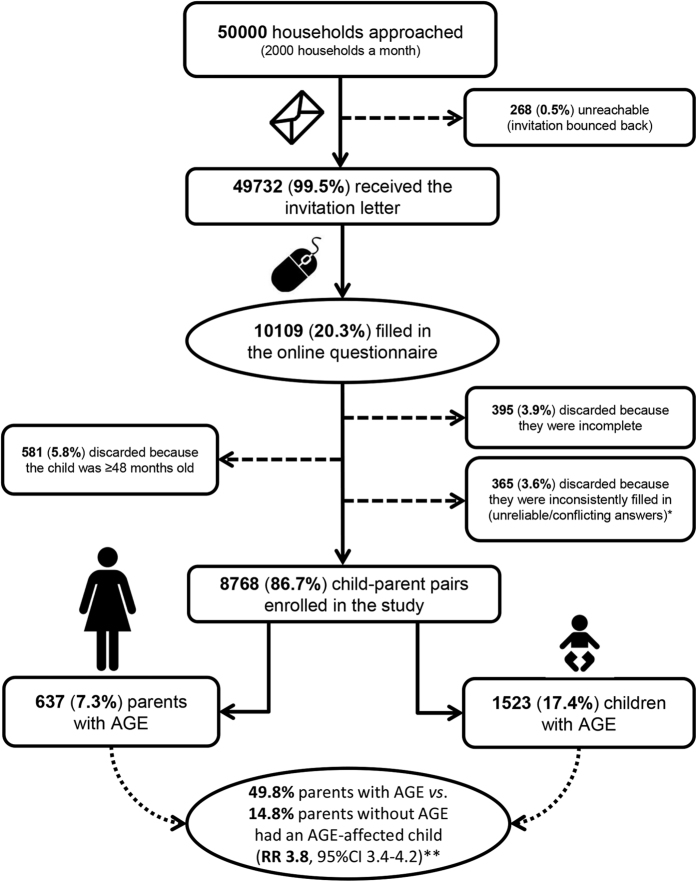
Flowchart of the sampling design. AGE = acute gastroenteritis; RR = risk ratio; 95%CI = 95% confidence interval. *The most common unreliable/conflicting answers leading to the questionnaire being discarded were those denoting mixing up of answers for the child with those for the parent and vice versa, or when the parents had reported data for a child other than the one invited to participate. Other examples of unreliable data were, for instance, reporting to be male and pregnant, being too young to be a parent (e.g. 10 year old), evident mistakes in reporting the date of birth (e.g. being born in the 1800s), reporting to have a partner living in the household but that only 1 adult lived in the household, reporting that no children lived in the household, etc. **Adjusted for the variables presented in Table 3.

Monthly, a random sample of 2000 <4-year-old children living in different households was drawn and their parents were invited by regular mail to complete a web-based questionnaire for the sampled child and for one parent chosen freely (Fig. 1). Sample size was based on 5% α-level, 1% precision, 22% expected AGE monthly incidence in <4-year-old children^3^, a population of 653,165 <4-year-old children in the participating municipalities as of 1^st^ June 2012, and an expected response rate of 13%, i.e. half of what can be expected from similar paper-and-pencil surveys^3^. The parent was informed on the main purpose of the survey, which was not limited to AGE, but generally aimed to assess the ‘health status’ of families with young children. Indeed, also other health conditions and aspects of family living standards were surveyed to complement data from current disease surveillance systems and population censuses. This study focused specifically on AGE given the still very high incidence of gastrointestinal infections in early childhood, their essentially preventable nature, and the limited published data on paediatric AGE outside the medical setting in developed countries. Upon completion of the questionnaire, parents gave consent also on behalf of the child. A given household was sampled only once, so only one child-parent pair per household participated.

The questionnaire was developed by expanding those used in previous population-based studies in the Netherlands^3^,^28^,^30^,^31^,^32^. Questions covered the prior four weeks and focused on household characteristics, DCC attendance, chronic diseases, medications, symptoms, medical care, absenteeism from work and from DCC, occupation, contact with animals, leisure activities, and eating habits (Supplementary Table S1). The urbanization degree and socio-economic status (SES) at the postcode level were obtained from Statistics Netherlands (http://www.cbs.nl/en-GB/menu/home/default.htm).

### Case Definition

We used a standard AGE case definition based on the symptoms reported in the questionnaire^33^. An AGE case was defined as a person with ≥3 diarrhoeal discharges or any ‘clinically relevant’ vomiting (in absence of pregnancy) in 24 hours during the previous four weeks, but excluding those cases of probable non-infectious origin, i.e. with underlying enteropathies. By ‘clinically relevant’ vomiting we refer to vomiting events other than regurgitation, vomiting due to motion sickness/vertigo, traumatic event, nauseous event (e.g. seeing others vomit), or drug/alcohol abuse. The exclusion criteria of pregnancy and underlying enteropathy, however, were not applied for the risk factor analysis, as these factors were always controlled for in the analysis (see below). Multiple episodes of AGE during the 4-week recall period were not differentiated and counted as one.

## Data Analysis

The societal burden of AGE was expressed as incidence, episodes/person-year (calculated as [365/28] × [4-weekly incidence proportion])^3^,^5^,^32^, medical care (general practitioner [GP] visits and hospitalizations), work and DCC days missed.

Logistic regression was used to identify factors associated with childhood and parental AGE. A total of 76 and 87 factors putatively associated with AGE in children and parents (Supplementary Table S1) was assessed in a ‘single-variable’ analysis including the following control covariates: child age group (infants, ≤12 months; toddlers, 13–36 months; pre-schoolers, 37–47 months) or parent age group (≤30, 31–34, 35–37, ≥38 years), gender, pregnancy, SES (low, intermediate, high), urbanization degree (<500, 500–1000, 1000–1500, 1500–2500, >2500 addresses/km2), season (Autumn, September–November; winter, December–February; spring, March–May; summer, June–August), year (2012–2014), and underlying enteropathies (e.g. bowel cancer, inflammatory bowel disease, irritable bowel syndrome, ulcerative colitis, celiac disease, Crohn’s disease, food allergy/intolerance, malabsorption syndromes, gastroesophageal reflux disease, chronic gastritis, and peptic ulcer disease). Variables with a p ≤ 0.10 were selected for inclusion in a multivariable model built in backward stepwise fashion, with variables showing a p < 0.05 being retained in the final model; the above covariates were always controlled for. The effect of removing variables on the other covariates was also monitored. Biologically plausible interactions were also tested as reported elsewhere^30^,^31^. Associations were expressed as risk ratios (RR) providing 95% confidence intervals (95%CI). Selection between collinear variables was made based on improved model fit as revealed by the Akaike information criterion (AIC). A complete record analysis was performed. Selection of covariates was theoretically informed, and variables to be tested were chosen based on previous studies, biological plausibility of being associated with AGE, and scientific interest of the research team. The variable ‘presence/absence of AGE in the participating child’ was then included as an additional explanatory variable in the multivariable model predicting parental AGE, allowing the association between AGE in the participating children and (their) parents to be tested. However, as it would not have been entirely correct to explain parental AGE with children’s AGE since we had no information on the AGE status of their non-participating siblings and other family members, risk factors for parental AGE were studied independently of children’s AGE. The final multivariable models showed an overall statistical significance (likelihood-ratio *χ*^2^-test, p < 0.05) and goodness-of-fit (Hosmer-Lemeshow test, p > 0.05). To cross-validate the inferences of the fitted models, bias-corrected bootstrap estimates were also calculated (1000 replications) and compared with the standard ones^34^. As these confidence intervals did not differ significantly, the standard ones were reported. For simplicity, only the final multivariable model results were presented. A more detailed description of the model-building approach is reported as Supplementary Information S1. Statistical analyses were performed using STATA 13 (StataCorp LP, College Station, TX, USA).

### Ethics Statement

This study conformed to the principles of the Declaration of Helsinki and received ethical approval by the Medical Research Ethics Committee of Utrecht University (WAG/om/13/048247). All methods were carried out in accordance with approved guidelines and regulations. Informed consent was obtained from all subjects. No subject-identifiable data were generated.

## Results

### Sample Characteristics

Overall, 10109 (20.3%) of the 49732 households receiving the invitation completed the questionnaire; 268 (0.5%) were unreachable (incomplete/changed address). After data cleaning (illustrated in Fig. 1), 8768 child-parent pairs were enrolled in the study. With regard to child’s age and gender, season, SES, and urbanization degree, for which we had information for both participants and non-participants, our child-parent pairs were representative of the target sample for child’s age, gender, and season, but there were significant differences for SES and urbanization degree (Supplementary Table S2). Specifically, the survey tended to underrepresent households from highly urbanized areas and from areas with high SES, and to overrepresent those from lowly urbanized/rural areas and from areas with low SES. To address this, we applied a weighting adjustment by SES and urbanization degree so that the incidence estimations accounted for imperfect representativity of the sample by weighting it back to the population from which it was drawn.

Questionnaires were completed by 7268 mothers (median age 34 years, interquartile range [IQR] 31–37) and 1500 fathers (37, 33–41), matching with 4210 female (median age 27 months, IQR 16–37) and 4558 male (26, 16–38) children. DCCs were attended by 50.4% of the children for a median of two days/week (IQR 2–4). Median duration of DCC attendance was 24 months (IQR 9–24). Most families (98.0%) had two parents, and had two children (47.2%), followed by those with one child (35.0%) and ≥3 children (17.8%).

### Burden

A total of 637 (7.3%) parents and 1523 (17.4%) children experienced AGE, corresponding to a (weighted) incidence of 0.95 episodes/parent-year (95%CI 0.88–1.02) and 2.25 episodes/child-year (2.15–2.36). In 317 (3.6%) child-parent pairs, both parent and child experienced AGE in the same 4-week period. Since our sample overrepresented mothers relative to fathers, sex-standardized parental incidence was also calculated: 6.5%, i.e. 0.85 episodes/parent-year (95%CI 0.77–0.94).

**Figure 2:**
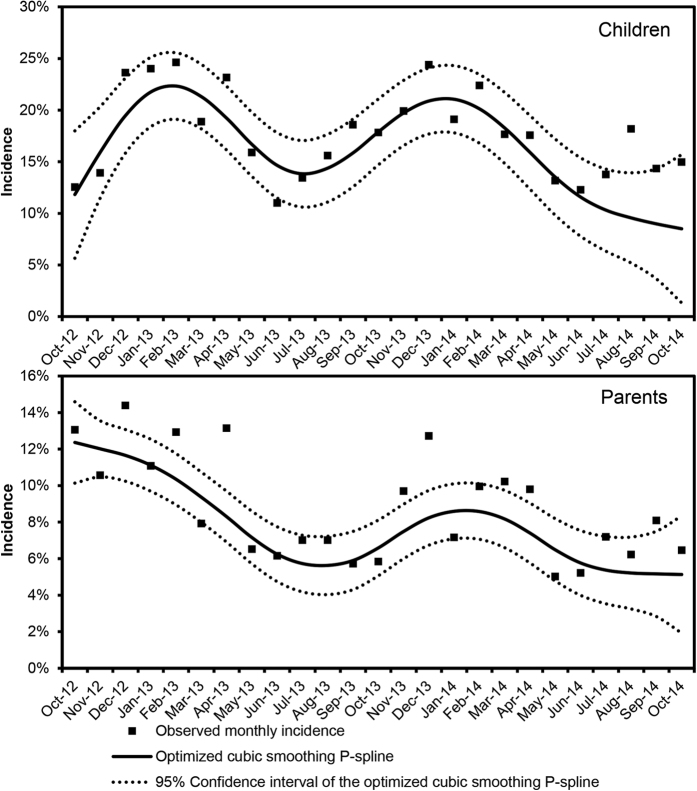
Incidence of acute gastroenteritis in children of <4 years of age and in their parents (n = 8768 child-parent pairs) by study month. An optimized cubic smoothing P-spline function is fitted to the observed data. Autumn, September–November; winter, December–February; spring, March–May; summer, June–August.

Monthly AGE incidence varied from 3.5–12.9% in parents to 11.0–26.3% in children, peaking in winter and decreasing in summer (Fig. 2). Of the 637 parent-cases, 22.8% had diarrhoea and vomiting, 47.7% only diarrhoea, and 29.1% only vomiting. Of the 1523 child-cases, 20.9% had diarrhoea and vomiting, 32.2% only diarrhoea, and 46.9% only vomiting; additional symptom descriptives are reported in the Supplementary Table S3. AGE lasted for a median of three days in both parents (IQR 2–4) and children (1–5). GP utilization was 18.3% for child cases and 8.6% for parent cases, and 1.6% of child cases but no parents were hospitalized. Most hospitalized children were toddlers (54.2%) and infants (37.5%), and attended DCCs (64.3%). Absenteeism from work was reported by 29.8% of parent cases and (by the parents of) 15.8% of child cases. Additionally, for 7.2% and 2.8% of parent and child cases, respectively, other people than the parents had to miss work. Child DCC absenteeism was 26.2% (Table 1).

### Risk Factors

Significant risk factors for childhood AGE in the final multivariable model including 1536 AGE-affected and 7232 AGE-free children are reported in Table 2. These were having underlying gastrointestinal, respiratory, or developmental conditions, using gastric antacids, having the participating parent employed in healthcare, living in a single-parent family, and having multiple siblings. Compared to home-cared children, those attending DCCs were at increased risk of AGE until twelve months of attendance, but afterwards this risk was not significant. Several animals in/around the households were tested for association with AGE (Supplementary Table S1), but only ownership of poultry/birds was significant. There was an interaction between child age and breastfeeding: breastfed infants of ≤6 months of age (but not those aged >6 months) had a significantly lower AGE risk than the never-breastfed infants, against which toddlers had an increased AGE risk (Table 2).

Table 3 shows the factors independently associated with parental AGE in the final multivariable model based on 764 AGE-affected and 8004 AGE-free parents. These were female gender, having underlying enteropathies, using gastric antacids, having a DCC-attending developmentally-disabled child, having multiple DCC-attending children, using antimicrobials in autumn/winter for pre-existing conditions other than AGE, primary consumption of meat bought directly from farmers, and having to normally wait for >1 hour between grocery shopping and food refrigeration. Older age and cleaning the fridge weekly were protective (Table 3). Parents whose participating children had AGE were at increased risk for AGI (RR 3.77, 95%CI 3.38–4.19).

## Discussion

AGE was estimated at 2.26 episodes/child-year and 0.95 episodes/parent-year, in line with the last Dutch community-based survey (2009–2010) employing a comparable case definition^3^. However, we found higher rates of GP visits (18.6% for children and 8.6% for parents) and hospitalization (1.6%) compared to those observed previously (GP visits: 12.8% for ‘children’, *n* = 39, and 5.7% for ‘adults’, *n* = 105; hospitalization: 0.7%, *n* = 146)^3^. An explanation may be the longer duration of our AGE episodes, as 50% of all our cases *vs*. 29% (*n* = 133) of the previous ones had symptoms for ≥3 days (Table 1) and were therefore likely to contact a physician^3^,^10^,^12^. This suggests a higher severity of AGE in families with young children, as also supported by the higher absenteeism from work (37% for parental AGE and 18.6% for childhood AGE in our survey *vs*. 14.4% among all 146 AGE cases found previously) and from DCC (26% *vs*. 13.7%)^3^.

AGE seasonality was similar between children and parents (Fig. 2), whereas in surveys that did not specifically focus on parents, childhood AGE seasonality differed considerably from that of the rest of the (adult) population^9^,^10^, as it mainly reflected the age-specific pathogen distribution in children and adults throughout the year^35^,^36^. Our finding is therefore suggestive of common aetiological agents, particularly viruses, shaping the observed seasonal patterns, indicating either shared sources of infection or household transmission. The strong association between AGE occurrence in children and (their) parents supports this hypothesis. However, because the onset of illness in children and parents was not available, we could not determine whether household transmission occurred, nor if it had any directionality, though even this would not have provided conclusive evidence. Nevertheless, previous research showed that parents frequently acquire AGE in secondary transmission from their children^7^,^8^. Our finding that mothers were more likely to experience AGE than fathers supports this notion, as previous studies suggested that the traditionally female-oriented task of preparing food^15^,^37^ and caring for the children^10^,^12^,^15^ may place women at increased exposure to enteropathogens than men.

Some pathogens known to cause foremost respiratory infections may also cause AGE symptoms and vice versa^38^,^39^. When excluding AGE cases with concurrent respiratory symptoms (cough, sore throat, runny nose, or breathing difficulties), our AGE incidence decreased by 55.4% in children and 30.0% in parents (results not shown). Similar estimates were reported in other surveys^10^,^12^,^39^, suggesting that respiratory pathogens may contribute to the AGE burden.

Alike all retrospective surveys, recall bias and specifically ‘telescoping’, i.e. when people remember disease episodes as being more recent than they actually are, may have occurred, leading to an overestimation of AGE incidence^26^. However, recall periods of ≥30 days may even lead to an underestimation of AGE incidence as respondents may forget (mild) illness episodes^13^. The somewhat low response rate (20.3%), although comparable to other studies^9^,^11^,^22^, may have represented a potential source of bias, as people who suffered from AGE might have been more motivated to participate. However, response rates were not particularly high in winter (Supplementary Table S2) when AGE incidence was highest (Fig. 2). Finally, we cannot exclude that some misclassification of AGE cases due to non-infectious causes might have occurred.

Breastfeeding was confirmed to reduce AGE risk in the first six months of life compared to never-breastfed infants^40^. The increased risk for toddlers *vs*. infants is probably related to their changed feeding habits and start of active interactions with peers and surroundings. Having underlying enteropathies and taking gastric antacids, especially proton-pump inhibitors, have previously been associated with AGE^3^ and specific bacterial infections like salmonellosis^31^ and campylobacteriosis^30^. It is conceivable that a chronically disturbed digestive tract may facilitate infections and that neutralization of gastric acidity may favour a pathogen’s survival during passage through the stomach. Increased AGE occurrence in asthmatic children has also been documented^3^,^41^, though the reasons remain unclear.

We found that children with developmental disabilities had an increased AGE risk. Previous research showed that children with autism-spectrum disorders have an 8-fold increased risk for AGE than non-disabled children^42^. The underlying reasons might be that these children more often display at-risk behaviours, such as suboptimal hygienic habits and extreme food selectivity. Our study indicated that also the parents of (DCC-attending) disabled children were at increased risk for AGE, possibly reflecting a higher chance of secondary transmission, especially since these children might need extra/special assistance for personal hygiene.

Parental occupation in healthcare was a risk factor for childhood AGE. Although this may suggest pathogen carriage from a parent’s work setting to the household, it is also possible that parents employed in healthcare, by virtue of their knowledge on health-related topics, reported more thoroughly. Challenges in continual supervision of a child’s hygiene behaviours and nurture needs due to likely parental task overload and recourse to various ‘outsourced’ childcare arrangements might explain the higher risk in children of single-parent families, while increased opportunities for household transmission would explain the higher risk associated with multiple siblings. Interestingly, of all the animals kept in/around the household we tested for, only poultry/birds were significant, warranting further attention on this exposure.

Compared to home-cared children, those attending DCCs were at increased risk of AGE for the first year of attendance, but not beyond, suggesting that immunological maturation may result from the (excess of) infections experienced during that first year of attendance (since we adjusted for child’s age), as also suggested by a recent Danish study^43^. Prior exposure and increased immunological maturity in our parents (i.e. young adults) may also explain why older parents experienced less AGE.

Parents were at increased risk of AGE with increasing number of DCC-attending children, confirming that attending DCCs does not only pose a risk to children, but also to parents via increased secondary transmission^7^. Another risk factor for parental AGE was using antimicrobials in autumn/winter. Antimicrobial-induced disturbance of the gut microbiome in a period of high pathogen activity would reduce intrinsic resistance to clinically overt infections. While viral aetiology seems plausible given the seasons in question and that taking antibiotics has been reported to protect against salmonellosis^31^ and campylobacteriosis^30^, antimicrobial-mediated depletion of normal gut microbiota can also diminish enteric virus replication^44^. Yet, *Clostridium difficile* infections, which seasonal peak occurs during autumn/winter, have increasingly been related to antimicrobial use^45^. Lastly, we pointed out that buying meat directly from farmers, delays in food refrigeration, and infrequent fridge cleaning increase parental AGE risk.

In conclusion, AGE occurs frequently in families with preschool children, causing high healthcare-seeking behaviours and productivity losses. Similar seasonality, strong association between parental and childhood AGE, and increased maternal risk suggest extensive household transmission of AGE-causing agents. The identified risk factors, some of which are in principle modifiable (i.e. food safety practices, medication use, breastfeeding, animal exposure), might provide targets for AGE-reducing initiatives in the surveyed population. These should focus on improving child-rearing hygiene, targeting mainly mothers, families with multiple siblings, with developmentally-disabled children, single parents, and with parents working in healthcare. A particular target should be DCCs, as they were a major determinant of AGE in both children and parents. However, the increased risk amongst DCC attendees lasted until one year of attendance, suggesting a gradual acquisition of immunity. This is a reassuring finding given the increased reliance on DCCs in high-income countries due to growing employment of women and rise in single-parent households. Efforts are nonetheless needed to improve hygiene in DCCs as to prevent pathogen transmission.

## Additional Information

**How to cite this article**: Mughini-Gras, L. *et al.* Societal Burden and Correlates of Acute Gastroenteritis in Families with Preschool Children. *Sci. Rep.* **6**, 22144; doi: 10.1038/srep22144 (2016).

## Supplementary Material

Supplementary Information

## Figures and Tables

**Table 1:** Burden of acute gastroenteritis in parents and children.

	Parents with AGE (*n *= 637)	Children with AGE (*n *= 1523)
Contacted the general practitioner	55 (8.6%)	278 (18.3%)
Hospitalized	None	25 (1.6%)
Parent(s) absent from work	190 (29.8%)	241 (15.8%)
median days of absence (IQR)	1.5 (1–2)	1 (1–2)
Others than the parent(s) absent from work	46 (7.2%)	42 (2.8%)
median days of absence (IQR)	1 (1–1)	1 (1–1)
Child absent from day-care	-	222/847* (26.2%)
median days of absence (IQR)	-	1 (1–2)
Median duration of illness in days (IQR)	3 (2–4)	3 (1–5)
Medication use**	100 (15.7%)	135 (8.9%)

IQR*** *****= **Interquartile range; AGE*** *****= **acute gastroenteritis

^*^Number of AGE child cases attending day-care centres.

^**^Antidiarrhoeals, antiemetics, antimicrobials, antispasmodic, antipyretic, and anti-inflammatory drugs.

**Table 2:** Multivariable risk ratios, corresponding 95% confidence intervals and adjusted predictions of the factors significantly associated with acute gastroenteritis in children of <4 years of age.

	Children with AGE (*n* = 1536)	Children without AGE (*n* = 7232)	Adjusted RR^1^ (95%CI)	Adjusted AGE predictions^1^,^7^
Season^2^				
Summer	283 (18.42%)	1765 (24.41%)	Reference	13.56%
Spring	410 (26.69%)	1899 (26.26%)	1.28 (1.12–1.45)	17.44%
Autumn	396 (25.78%)	2066 (28.57%)	1.21 (1.05–1.39)	16.53%
Winter	447 (29.10%)	1502 (20.77%)	1.67 (1.48–1.88)	23.08%
Child age^3^ x breastfeeding				
Never-breastfed infant	64 (4.17%)	352 (4.87%)	Reference	14.08%
≤6 month-old breastfed infant	19 (1.24%)	245 (3.39%)	0.51 (0.31–0.84)	7.22%
>6 month-old breastfed infant	53 (3.45%)	232 (3.21%)	1.29 (0.92–1.74)	18.21%
Infant with unknown breastfeeding history	130 (8.46%)	531 (7.34%)	1.31 (0.99–1.69)	18.57%
Toddler	913 (59.44%)	3886 (53.73%)	1.34 (1.06–1.66)	18.93%
Pre-schooler	357 (23.24%)	1986 (27.46%)	1.13 (0.87–1.44)	15.96%
Chronic enteropathies^4^				
No	1352 (88.02%)	6786 (93.83%)	Reference	16.82%
Yes	184 (11.98%)	446 (6.17%)	1.55 (1.32–1.79)	25.80%
Chronic respiratory diseases^5^				
No	1388 (90.36%)	6853 (94.76%)	Reference	17.04%
Yes	148 (9.64%)	379 (5.24%)	1.44 (1.23–1.68)	24.33%
Developmental disabilities^6^				
No	1477 (96.16%)	7118 (98.42%)	Reference	17.26%
Yes	59 (3.84%)	114 (1.58%)	1.72 (1.35–2.15)	29.26%
Using gastric antacids				
No	1488 (96.88%)	7152 (98.89%)	Reference	17.35%
Yes	48 (3.13%)	80 (1.11%)	1.56 (1.13–2.09)	26.77%
Parent working in healthcare				
No	1148 (74.74%)	5611 (77.59%)	Reference	17.04%
Yes	388 (25.26%)	1621 (22.41%)	1.13 (1.01–1.25)	19.11%
Single-parent family				
No	1494 (97.27%)	7101 (98.19%)	Reference	17.40%
Yes	42 (2.73%)	131 (1.81%)	1.35 (1.01–1.76)	23.25%
*N* children in the house				
1 child	492 (32.03%)	2574 (35.59%)	Reference	15.73%
2 children	770 (50.13%)	3370 (46.60%)	1.19 (1.07–1.32)	18.69%
≥3 children	274 (17.84%)	1288 (17.81%)	1.15 (1.00–1.31)	17.99%
Cumulated DCC attendance				
None	680 (44.27%)	3670 (50.75%)	Reference	15.79%
1–3 months	72 (4.69%)	275 (3.80%)	1.47 (1.18–1.81)	23.25%
4–6 months	83 (5.40%)	250 (3.46%)	1.51 (1.22–1.84)	23.86%
7–12 months	291 (18.95%)	1029 (14.23%)	1.33 (1.18–1.51)	21.04%
13–24 months	246 (16.02%)	1094 (15.13%)	1.11 (0.97–1.27)	17.56%
>24 months	164 (10.68%)	914 (12.64%)	1.03 (0.86–1.22)	16.20%
Owning poultry and/or birds				
No	1406 (91.54%)	6724 (92.98%)	Reference	17.25%
Yes	130 (8.46%)	508 (7.02%)	1.23 (1.04–1.44)	21.05%

AGE = acute gastroenteritis; RR = risk ratio; 95%CI = 95% confidence interval; DCC = day-care centre.

^1^Adjusted for urbanization degree, socio-economic status, year, and child’s gender in addition to all the other variables included in this table.

^2^Autumn, September–November; winter, December–February; spring, March–May; summer, June–August.

^3^Infant, ≤12 months; toddler, 13–36 months; pre-schooler, 37–47 months.

^4^E.g. bowel cancer, inflammatory bowel disease, irritable bowel syndrome, ulcerative colitis, celiac disease, Crohn’s disease, food allergy/intolerance, malabsorption syndromes, gastroesophageal reflux disease, chronic gastritis, and peptic ulcer disease.

^5^E.g. asthma, chronic obstructive pulmonary disease and other chronic lung diseases, respiratory allergies, lung cancer, and pulmonary hypertension.

^6^E.g. mental retardation, cerebral palsy, autism spectrum disorders, attention-deficit/hyperactivity disorder, Down’s syndrome and other genetic disorders, congenital defects, learning disabilities, mental illness, and traumatic brain injury.

^7^Aka predictive margins are the adjusted prevalences of AGE for each stratum of the independent variables included in the model and denote the probability for an AGE event to occur for individuals in those strata.

**Table 3:** Multivariable risk ratios, corresponding 95% confidence intervals and adjusted predictions of the factors significantly associated with acute gastroenteritis in parents of children of <4 years of age.

	Parents with AGE (*n* = 764)	Parents without AGE (*n* = 8004)	Adjusted RR^1^ (95%CI)	Adjusted AGEpredictions^1^,^5^
Season^2^				
Summer	134 (17.54%)	1914 (23.91%)	Reference	6.97%
Spring	198 (25.92%)	2111 (26.37%)	1.33 (1.08–1.63)	9.27%
Autumn	214 (28.01%)	2248 (28.09%)	1.13 (0.90–1.41)	7.88%
Winter	218 (28.53%)	1731 (21.63%)	1.57 (1.28–1.92)	10.93%
Parent age				
≤30 years	160 (20.94%)	1325 (16.55%)	Reference	11.07%
31–34 years	255 (33.38%)	2457 (30.70%)	0.84 (0.68–1.02)	9.35%
35–37 years	181 (23.69%)	1845 (23.05%)	0.77 (0.62–0.96)	8.69%
≥38 years	168 (21.99%)	2377 (29.70%)	0.59 (0.47–0.74)	6.68%
Parent gender				
Male	92 (12.04%)	1408 (17.59%)	Reference	6.56%
Female	672 (87.96%)	6596 (82.41%)	1.39 (1.13–1.7)	9.13%
Chronic enteropathies^3^				
No	672 (87.96%)	7627 (95.29%)	Reference	8.16%
Yes	92 (12.04%)	377 (4.71%)	2.18 (1.76–2.67)	17.56%
Using gastric antacids				
No	747 (97.77%)	7950 (99.33%)	Reference	8.64%
Yes	17 (2.23%)	54 (0.67%)	1.82 (1.08–2.94)	15.47%
Using antimicrobials for conditions other than AGE				
No	732 (95.81%)	7880 (98.45%)	Reference	8.55%
Yes, in autumn/winter	17 (2.23%)	53 (0.66%)	2.15 (1.31–3.36)	18.02%
Yes, in spring/summer	15 (1.96%)	71 (0.89%)	1.65 (0.97–2.69)	13.94%
N children in the house attending DCCs				
None	296 (38.74%)	3640 (45.48%)	Reference	7.29%
1 child	285 (37.30%)	2944 (36.78%)	1.26 (1.07–1.47)	9.17%
2 children	168 (21.99%)	1325 (16.55%)	1.54 (1.28–1.85)	11.22%
≥3 children	15 (1.96%)	95 (1.19%)	1.95 (1.19–3.07)	14.18%
Having a child with developmental disabilities^4^				
No	738 (96.60%)	7857 (98.16%)	Reference	8.60%
Yes, attending a DCC	18 (2.36%)	60 (0.75%)	1.03 (0.51–1.97)	19.00%
Yes, not attending a DCC	8 (1.05%)	87 (1.09%)	2.27 (1.42–3.43)	8.84%
Primary type of meat consumed				
No meat consumption	26 (3.40%)	343 (4.29%)	Reference	7.88%
Regular meat from butcher/supermarket	629 (82.33%)	6572 (82.11%)	1.10 (0.75–1.58)	8.64%
Meat directly from farmers	32 (4.19%)	210 (2.62%)	1.69 (1.03–2.66)	13.20%
Organic meat	77 (10.08%)	879 (10.98%)	1.07 (0.70–1.62)	8.45%
Cleaning frequency of the fridge				
Less often than once a month	551 (72.12%)	5697 (71.18%)	Reference	8.88%
Every month	175 (23.04%)	1781 (22.25%)	1.00 (0.84–1.18)	8.89%
Every week	37 (4.84%)	526 (6.57%)	0.70 (0.50–0.98)	6.32%
Average time between grocery shopping and refrigeration				
<1 hour	429 (56.15%)	4841 (60.48%)	Reference	8.16%
1–2 hours	296 (38.74%)	2847 (35.57%)	1.15 (1.00–1.33)	9.39%
>2 hours	39 (5.10%)	316 (3.95%)	1.35 (0.98–1.83)	10.93%

AGE = acute gastroenteritis; RR = risk ratio; 95%CI = 95% confidence interval; DCC = day-care centre.

^1^Adjusted for urbanization degree, socio-economic status, year, and pregnancy status in addition to all the other variables included in this table.

^2^Autumn, September–November; winter, December–February; spring, March–May; summer, June–August.

^3^E.g. bowel cancer, inflammatory bowel disease, irritable bowel syndrome, ulcerative colitis, celiac disease, Crohn’s disease, food allergy/intolerance, malabsorption syndromes, gastroesophageal reflux disease, chronic gastritis, and peptic ulcer disease.

^4^E.g. mental retardation, cerebral palsy, autism spectrum disorders, attention-deficit/hyperactivity disorder, Down’s syndrome and other genetic disorders, congenital defects, learning disabilities, mental illness, and traumatic brain injury.

^5^Aka predictive margins are the adjusted prevalences of AGE for each stratum of the independent variables included in the model and denote the probability for an AGE event to occur for individuals in those strata.
