# Supplementary material for: Societal Burden and Correlates of Acute Gastroenteritis in Families with Preschool Children
Source: Sci Rep. 2016 Feb 26;6:22144. doi: 10.1038/srep22144 (PMC4768267; doi:10.1038/srep22144)
Supplement: Supplementary Information [file srep22144-s1.pdf]

Societal Burden and Correlates of Acute Gastroenteritis in Families with Preschool Children

Lapo Mughini-Gras, Roan Pijnacker, Moniek Heusinkveld, Remko Enserink, Rody Zuidema, Erwin Duizer, Titia Kortbeek, Wilfrid van Pelt

---

Supplementary Information S1. Details of the model-building approach used in the risk factor analysis for acute gastroenteritis in preschool children and their parents.

Risk factors for acute gastroenteritis (AGE) in preschool children and their parents were studied using logistic regression models, which are presented in detail in more specialized sources[1]. In our study, the dichotomous outcome variable was having had AGE (encoded as 1) or not (encoded as 0). Two separate multivariable models were built, one for the children and the other one for the parents. In total, 76 (for children) and 87 (for parents) putative risk factors (see Supplementary Table S1) were tested as independent variables for association with the outcome variable. The choice of these variables was theoretically informed on the basis of previous studies, biological plausibility of being associated with AGE, and scientific interest of the research team. Our goal was to find the best-fitting, biologically sound and parsimonious model describing the relationship between the outcome of interest (i.e. AGE, aka dependent or response variable) and the aforementioned set of independent (aka predictor or explanatory) variables. Logistic regression estimated the coefficients, their uncertainty and significance levels based on the logit transformation of the probability of experiencing AGE. The basic formula of the logistic regression model with *n* candidate predictors used in this study was as follows:

$$logit(p) = \ln\left(\frac{p}{1-p}\right) = b_0 + b_1X_1 + b_2X_2 + b_3X_3 \ldots + b_nX_n$$

where p is the probability of experiencing AGE, b_0 is the constant (intercept) term, and b_1, b_2, b_3 ... b_n are the regression coefficients of the independent variables X_1, X_2, X_3 … X_n, which estimated the increase/decrease in the log odds of the outcome per unit increase in the value of the predictor.

We followed a standard model-building procedure that is described in several (textbook) sources[2,3], and has been used in several previous AGE-themed studies, e.g.[4-12]. In brief, the regression models were built using a stepwise variable selection approach. First, for preliminary significance testing, a 'single-variable' analysis was performed in which each independent variable of interest was tested for association with the outcome in a separate model including also, as explanatory

variables, the following *a priori* potential confounders: child or parent age group and gender, pregnancy, socioeconomic status, urbanization degree, sampling season and year, and underlying enteropathies. Variables whose associations with the outcome showed a *p*-value ≤0.10 for the null hypothesis that the coefficient was 0 were selected for entry in a multivariable logistic regression model to sort out those variables independently associated with the outcome, while forcing the *a priori* confounders first. The multivariable model was built in backward stepwise fashion, meaning that all variables selected at the 'single-variable' analysis were entered in the model and then dropped one by one, starting from the least significant one, until all variables in the model showed a *p*-value <0.05. Variable selection involved starting off in a backward manner, but then all dropped variables (except the most recently dropped) were reconsidered for (forward) re-entry into the model if they later appeared to be significant following their order of drop off and using a more stringent significance level for being added back into the model (*p*-value <0.01). The above *a priori* confounders were always controlled for. The effect of removing/adding variables on the other predictors was strictly monitored, with a change of ≥10% in the coefficients of the other predictors being considered as a sign of confounding, so that the variables in question were retained in the model regardless of their significance. To avoid problems of multicollinearity, i.e. when two or more predictors are highly correlated with each other affecting the reliability of parameter estimates, collinear variables were identified prior to multivariable analysis by looking at their (pairwise) covariance matrix. Selection between collinear variables was based on the variables that improved model fit the most as revealed by the lowest Akaike information criterion (AIC) values, or on biological plausibility, reliability and number of observations when the collinear variables measured similar factors[3]. Biologically plausible interactions between independent variables were first tested for significance in the 'single-variable' analysis and then in the multivariable models, which were then expanded to include the significant interaction terms.

After identifying the final multivariable model for AGE in parents, we tested weather the parents of AGE-affected children were at increased risk of experiencing AGE concurrently with their children. To this goal, the variable 'presence/absence of AGE in the participating child', i.e. the dependent variable of the model for children, was entered as an additional explanatory variable in the final multivariable model of parental AGE. This allowed the association between AGE in the enrolled children and AGE in their participating parents to be tested. However, as we had no information on the

AGE status of the household members other than the enrolled children, it would not have been entirely correct to explain parental AGE with the children's AGE. This is why the risk factors for parental AGE were studied independently of children's AGE.

The regression coefficients of the final multivariable models were exponentiated to obtain the odds ratio (OR) for each independent variable. ORs are generally used to compare the relative odds of the occurrence of the outcome given the exposure to the variable of interest. However, because the prevalence of the outcome was common in our study population, the logistic regression OR would no longer approximate the risk ratio (RR), which is a better indicator of the strength of associations as it denotes the ratio of the risk of AGE occurrence among the exposed to that among the unexposed. As the higher the prevalence of the outcome is, the more the ORs over-estimate the RRs when greater than 1, and the more they under-estimate the RRs when under 1, Zhang and Yu[13] proposed a simple method for adjusting the (multivariable) logistic regression ORs so that they approximate the RRs. The formula for expressing RRs in terms of the ORs is as follows:

$$RR \sim \frac{OR}{(1 - pu) + (pu \ \times OR)}$$

where *pu* is the prevalence rate for the unexposed to the variable of interest. We therefore converted the logistic regression ORs (and corresponding confidence intervals) to RRs using this method, which is implemented in the statistical software we used (STATA v.13).

A complete record analysis was performed, meaning that there were no missing values to be handled in the data set. Overall statistical significance and goodness-of-fit of the final multivariable models were tested with the likelihood-ratio χ^2 test and the Hosmer-Lemeshow test, respectively. Finally, to cross-validate the inference of the fitted models, bias-corrected bootstrap estimates were calculated (1000 replications) and compared with the standard ones. The procedure consisted of drawing 1000 random samples with replacement from the observed data and using these samples to feed back the models. Bootstrap 95% confidence intervals were calculated based on the model parameters at each replication; the bias statistic denoted how much each model parameter from the bootstrap distribution deviated from the parameter of the fitted models. Bias-corrected 95% CIs were then calculated so that the statistical significance of each parameter could be assessed in light of the fitted models applied to 'different' data, albeit drawn from the same population. This allowed us to examine the generalizability of the fitted models in order to cross-validate the inferences of the fitted models[14].

Supplementary Table S1. Variables tested for association with acute gastroenteritis.

**'Household-related' variables** (tested for both parents and children)	**Frequency of participants with that factor (*n*=8768)**
Family structure	
2 parents in the household	8595 (98.0%)
1 parent in the household	173 (2.0%)
1 or more dogs in the household (yes/no)	1465 (16.7%)
1 or more cats in the household (yes/no)	2246 (25.6%)
1 or more pet rodents and/or rabbits in the household (yes/no)	958 (10.9%)
1 or more cattle in the household (yes/no)	147 (1.7%)
1 or more horses in the household (yes/no)	218 (2.5%)
1 or more sheep in the household (yes/no)	134 (1.5%)
1 or more poultry and/or other birds (yes/no)	638 (7.3%)
1 or more pigs in the household (yes/no)	49 (0.6%)
1 or more goats in the household (yes/no)	93 (1.1%)
1 or more ornamental/aquarium fish in the household (yes/no)	198 (2.3%)
1 or more pet reptiles and/or amphibians in the household (yes/no)	23 (0.3%)
1 or more wild animals in the household (yes/no)	21 (0.2%)
Number of children (minors) living in the house	
1 child	3066 (35.0%)
2 children	4140 (47.2%)
≥3 children	1562 (17.8%)
Number of children attending day-care centres living in the house	
0 children	3936 (44.9%)
1 child	3229 (36.8%)
2 children	1493 (17.0%)
≥3 children	110 (1.3%)
Number of adults (over age 18) living in the house	
1 adult	173 (2.0%)
2 adults	8475 (96.7%)
≥3 adults	120 (1.4%)
Any smoker in the house (yes/no)	303 (3.5%)
Sandbox playground in the household (yes/no)	4589 (52.3%)
Highest education degree in the household	
primary, lower vocational or lower secondary education	547 (6.2%)
intermediate vocational, intermediate/higher secondary education	2370 (27.0%)
higher vocational, college and university education	5851 (66.7%)
Household in proximity to a woody area	801 (9.1%)
Household in proximity to an urban park	1154 (13.2%)
Household in proximity to a meadow	1932 (22.0%)
Household in proximity to a cropland	974 (11.1%)
Any vegetarian in the house	
0 vegetarians	8399 (95.8%)
≥1 vegetarians	369 (4.2%)
Cleaning frequency of the fridge	
<once/month	6248 (71.3%)
monthly	1957 (22.3%)
weekly	563 (6.4%)
Using the same chopping board for raw meat and other foods (yes/no)	926 (10.6%)
Using the same knife for raw meat and other foods (yes/no)	3265 (37.2%)
Material of the chopping board mainly used in the house	
wood	1577 (18.0%)
plastic	6391 (72.9%)
glass	796 (9.1%)
stone	4 (0.05%)
Cleaning kitchen cloths less than once a week (yes/no)	4749 (54.2%)
Average time between grocery shopping and food refrigeration	
<1 hour	5270 (60.1%)
1–2 hours	3143 (35.9%)
>2 hours	355 (4.1%)
Any household member went to a gardening store (yes/no)	3225 (36.8%)
Any household member has gardened (yes/no)	5503 (62.8%)
Any household member stayed in a garden (yes/no)	4481 (51.1%)
Any household member stayed in a holiday house (yes/no)	1743 (19.8%)
Any household member went camping (yes/no)	577 (6.6%)
Any household member went to a golf field (yes/no)	150 (1.7%)

Any household member went swimming in open water (yes/no)	746 (8.5%)
Any household member visited a sauna/waterpark/swimming pool (yes/no)	3965 (45.2%)
Any household member showered/bathed at school, sport club, etc. (yes/no)	3150 (35.9%)
Any household member went to a carwash (yes/no)	1815 (20.7%)
Any household member used a high-pressure sprayer/nozzle (yes/no)	2469 (28.2%)
Any household member worked on the heating/plumbing system (yes/no)	478 (5.45%)
Season	
Winter (December-February)	1949 (22.2%)
Autumn (September-November)	2462 (28.1%)
Spring (March-May)	2309 (26.3%)
Summer (June-August)	2048 (23.4%)
Degree of urbanization	
>2500 addresses/km^2 (highly urbanized)	1102 (12.6%)
1500–2500 addresses/km^2 (urbanized)	1848 (21.1%)
1000–1500 addresses/km^2 (moderately urbanized)	1725 (19.7%)
500–1000 addresses/km^2 (lowly urbanized)	1960 (22.4%)
<500 addresses/km^2 (rural)	2133 (24.3%)
Socio-economic status	
Low	3074 (35.1%)
Intermediary	2953 (33.7%)
High	2741 (31.3%)
**‘Child-related’ variables** (tested only for children)	
Age group	
≤12 months (infant)	1626 (18.6%)
13–36 months (toddler)	4799 (54.7%)
37–47 months (preschooler)	2343 (26.7%)
Breastfeeding history	
Never breastfed	2616 (29.8%)
Breastfed for ≤6 months from birth	3855 (44.0%)
Breastfed for >6 months from birth	1884 (21.5%)
Unknown breastfeeding history	413 (4.7%)
Sex	
Male	4558 (52.0%)
Female	4210(48.0%)
Attending a day-care centre (yes/no)	4418 (50.4%)
Attending child-care arrangements other than day-care centres (yes/no)	3164 (36.1%)
Cumulated day-care centre attendance	
0 months (home-cared)	4350 (49.6%)
1–3 months	347 (4.0%)
4–6 months	333 (3.8%)
7–12 months	1320 (15.1%)
13–24 months	1340 (15.3%)
>24 months	1078 (12.3%)
Suffering from developmental disability (yes/no)	173 (2.0%)
Suffering from diabetes (yes/no)	2 (0.02%)
Suffering from (chronic) dermatological conditions (yes/no)	323 (3.7%)
Suffering from (chronic) renal conditions (yes/no)	8 (0.09%)
Suffering from (chronic) gastrointestinal conditions (yes/no)	630 (7.2%)
Suffering from (chronic) respiratory conditions (yes/no)	527 (6.0%)
Suffering from (chronic) cardiovascular conditions (yes/no)	61 (0.70%)
Suffering from cancer (yes/no)	2 (0.02%)
Suffering from immunological disorders (yes/no)	29 (0.33%)
Suffering from (chronic) osteoarthrological/muscular conditions (yes/no)	14 (0.16%)
Suffering from neurological conditions (yes/no)	28 (0.32%)
Having had blood/plasma transfusion in the last 3 months (yes/no)	5 (0.1%)
Using antimicrobials (yes/no)	451 (5.1%)
Using corticosteroid drugs (yes/no)	152 (1.7%)
Using respiratory system drugs (yes/no)	249 (2.8%)
Using cardiovascular system drugs (yes/no)	2 (0.02%)
Using gastrointestinal system drugs (yes/no)	89 (1.0%)
Using gastric antacids (yes/no)	128 (1.5%)
Using painkillers (yes/no)	11 (0.1%)
Participating parent working with children (yes/no)	1557 (17.8%)
Participating parent working in healthcare (yes/no)	2009 (22.9%)
Participating parent working with animals (yes/no)	277 (3.2%)
Participating parent working with raw meat (yes/no)	197 (2.3%)
Participating parent working with food (yes/no)	802 (9.2%)

Participating parent working in animal farming (yes/no)	120 (1.4%)
**'Parent-related' variables** (tested only for parents)	
Age group	
≤30 years	1485 (16.9%)
31–34 years	2712 (30.9%)
35–37 years	2026 (23.1%)
≥38 years	2545 (29.0%)
Sex	
Male	1500 (17.1%)
Female	7268 (82.9%)
Having Dutch nationality (yes/no)	8477 (96.7%)
Being born in the Netherlands (yes/no)	8189 (93.4%)
Being currently employed (yes/no)	7491 (85.4%)
Working with children (yes/no)	1557 (17.8%)
Working in healthcare (yes/no)	2009 (22.9%)
Working with animals (yes/no)	277 (3.2%)
Working with raw meat (yes/no)	197 (2.3%)
Working with food (yes/no)	802 (9.2%)
Working in animal farming (yes/no)	120 (1.4%)
Being pregnant (yes/no)	1573 (17.9%)
Suffering from diabetes (yes/no)	62 (0.7%)
Suffering from (chronic) dermatological conditions (yes/no)	105 (1.2%)
Suffering from (chronic) renal conditions (yes/no)	15 (0.2%)
Suffering from (chronic) hepatological conditions (yes/no)	6 (0.1%)
Suffering from (chronic) gastrointestinal conditions (yes/no)	469 (5.6%)
Suffering from (chronic) respiratory conditions (yes/no)	1391 (15.9%)
Suffering from (chronic) cardiovascular conditions (yes/no)	74 (0.8%)
Suffering from cancer (yes/no)	28 (0.3%)
Suffering from immunological disorders (yes/no)	99 (1.1%)
Suffering from (chronic) osteoarthrological/muscular conditions (yes/no)	140 (1.6%)
Suffering from neurological conditions (yes/no)	22 (0.3%)
Using antimicrobials (yes/no)	152 (1.7%)
Using corticosteroid drugs (yes/no)	117 (1.3%)
Using respiratory system drugs (yes/no)	469 (5.4%)
Using cardiovascular system drugs (yes/no)	60 (0.7%)
Using gastrointestinal system drugs (yes/no)	63 (0.7%)
Using gastric antacids (yes/no)	71 (0.8%)
Using painkillers (yes/no)	97 (1.1%)
Frequency of row/undercooked meat consumption	
Never	1680 (19.2%)
<once/week	4525 (51.6%)
1–3 times/week	2340 (26.6%)
>3 times/week	223 (2.5%)
Frequency of chicken consumption	
Never	464 (5.3%)
<once a week	2180 (24.9%)
1–3 times a week	5839 (66.6%)
>3 times a week	285 (3.3%)
Frequency of egg consumption	
Never	89 (1.0%)
<once a week	2378 (27.1%)
1–3 times a week	5822 (66.4%)
>3 times a week	479 (5.5%)
Frequency of seafood consumption	
Never	1045 (11.9%)
<once a week	4258 (48.6%)
1–3 times a week	3419 (40.0%)
>3 times a week	46 (0.5%)
Frequency of dairy consumption	
Never	53 (0.6%)
<once a week	102 (1.2%)
1-3 times a week	618 (7.1%)
>3 times a week	7995 (91.2%)
Frequency of fruit consumption	
Never	2 (0.02%)
<once a week	38 (0.3%)
1–3 times a week	269 (3.1%)

>3 times a week	8469 (96.6%)
Frequency of raw vegetable consumption	
Never	80 (0.9%)
<once a week	813 (9.3%)
1–3 times a week	3614 (41.2%)
>3 times a week	4261 (48.6%)
Primary type of meat consumed	
No meat consumption	369 (4.2%)
Regular meat from butcher/supermarket	7201 (82.1%)
Meat purchased directly from farmers	242 (2.8%)
Organic meat	956 (10.9%)
Primary type of fruit/vegetables eaten	
Regular fruit/vegetables from shop/supermarket	7943 (90.6%)
Organic fruit/vegetables from shop/supermarket	477 (5.4%)
Fruit/vegetables from own garden	176 (2.0%)
Fruit/vegetables directly from farmers	172 (2.0%)
Frequency of hand washing before preparing food	
Never	37 (0.4%)
Rarely	126 (1.4%)
Sometimes	617 (7.0%)
Most of the times	3158 (36.0%)
Always	4830 (55.1%)
Frequency of hand washing after handling raw meat	
Never	34 (0.4%)
Rarely	75 (0.9%)
Sometimes	287 (3.3%)
Most of the times	1255 (14.3%)
Always	7117 (81.2%)
Frequency of hand washing after toilet visit	
Never	9 (0.1%)
Rarely	44 (0.5%)
Sometimes	131 (1.5%)
Most of the times	839 (9.6%)
Always	7745 (88.3%)

Supplementary Table S2. Questionnaire response of the child-parent pairs enrolled in the study.

	Invited child-parent pairs	Participating child-parent pairs	P-value (χ^2 test) invited *vs*. participating child-parent pairs	Response rate
Child age group				
≤12 months (infant)	9653 (19.4%)	1626 (18.5%)	0.3947	16.8%
13-36 months (toddler)	26433 (53.2%)	4799 (54.7%)	0.0553	18.2%
37-47 months (preschooler)	13646 (27.4%)	2343 (26.7%)	0.4823	17.2%
Child sex				
♂	25657 (51.6%)	4558 (52.0%)	0.6185	17.8%
♀	24075 (48.4%)	4210(48.0%)	0.6318	17.5%
Urbanization degree				
>2500 addresses/km^2 (highly urbanized)	8121 (16.3%)	1102 (12.6%)	0.0016	13.6%
1500-2500 addresses/km^2 (urbanized)	11409 (22.9%)	1848 (21.1%)	0.0863	16.2%
1000-1500 addresses/km^2 (moderately urbanized)	9747 (19.6%)	1725 (19.7%)	0.9232	17.7%
500-1000 addresses/km^2 (lowly urbanized)	9976 (20.1%)	1960 (22.4%)	0.0211	19.7%
<500 addresses/km^2 (rural)	10479 (21.1%)	2133 (24.3%)	0.0011	20.4%
SES[1]				
Low	15680 (31.5%)	3074 (35.1%)	0.0001	19.6%
Intermediate	15213 (30.6%)	2953 (33.7%)	0.0009	19.4%
High	18838 (37.9%)	2741 (31.3%)	0.0000	14.6%
Season[4]				
Winter (December-February)	11916 (24.0%)	1949 (22.2%)	0.0834	16.4%
Autumn (September-November)	13910 (28.0%)	2462 (28.1%)	0.5406	17.7%
Spring (March–May)	11906 (23.9%)	2309 (26.3%)	0.9931	19.4%
Summer (June–August)	12000 (24.1%)	2048 (23.4%)	0.2465	17.1%
Total	49732 (100%)	8768 (100%)		17.6%

1. SES = socio-economic status, expressed as a score going from –4 to 4, based on income, occupation, and education level per postcode. Low, –4 to –0.40; intermediate, –0.41 to 0.30; high, 0.31 to 4, based on the tertiles of the SES distribution of the whole of the Netherlands.

Supplementary Table S3. Self-reported gastrointestinal symptoms in the child-parent pairs.

	Parents (*n*=8768)	Children (*n*=8768)
Vomiting	414 (4.7%)	1041 (11.9%)
Diarrhoea (≥3 diarrhoeal discharges in 24 h)	509 (5.8%)	820 (9.4%)
Nausea	610 (7.0%)	291 (3.3%)
Abdominal pain	870 (9.9%)	924 (10.5%)
Mucus in the stool	71 (0.8%)	135 (1.5%)
Blood in the stool	51 (0.6%)	28 (0.3%)
Discoloration in the stool	77 (0.9%)	286 (3.3%)
Acute gastrointestinal illness[1]	Parents with AGE (*n*=637)	Children with AGE (*n*=1523)
Vomiting	333 (52.3%)	1033 (67.8%)
Diarrhoea (≥3 diarrhoeal discharges in 24 h)	449 (70.5%)	809 (53.1%)
Nausea	285 (44.7%)	241 (15.8%)
Abdominal pain	301 (47.3%)	446 (29.3%)
Mucus in the stool	21 (3.3%)	90 (5.9%)
Blood in the stool	10 (1.6%)	13 (0.9%)
Discoloration in the stool	32 (5.0%)	165 (10.8%)

AGE = acute gastroenteritis; IQR = interquartile range

1. Defined as having ≥3 diarrhoeal discharges and/or any vomiting (in absence of pregnancy) in 24 h, but excluding those with underlying gastrointestinal diseases that may have caused these symptoms.

References

1 Hosmer, D. W. J., Lemeshow, S. & Sturdivant, R.X. *Applied Logistic Regression*. 3rd edn, (Wiley, 2013).

2 Hill T, L. P. *Statistics: Methods and Applications: a Comprehensive Reference for Science, Industry, and Data Mining.*, 832 (Tulsa, OK: StatSoft Inc., 2006).

3 Dohoo, I. R., Wayne, M. & Stryhn, H. E. *Veterinary Epidemiologic Research*. (Charlottetown PEI: University of Prince Edward Island, 2009).

4 Enserink, R., Mughini-Gras, L., Duizer, E., Kortbeek, T. & van Pelt, W. Risk factors for gastroenteritis in child day care. *Epidemiol Infect.* 10.1017/s0950268814003367, 1-14 (2015).

5 De Man, H. *et al.* Gastrointestinal, influenza-like illness and dermatological complaints following exposure to floodwater: a cross-sectional survey in The Netherlands. *Epidemiol Infect.* 10.1017/s0950268815002654 (2015).

6 Mughini Gras, L. *et al.* Risk factors for campylobacteriosis of chicken, ruminant, and environmental origin: a combined case-control and source attribution analysis. *PLoS One.* **7**, e42599 (2012).

7 Mughini-Gras, L. *et al.* Risk factors for human salmonellosis originating from pigs, cattle, broiler chickens and egg laying hens: a combined case-control and source attribution analysis. *PLoS One.* **9**, e87933 (2014).

8 Doorduyn, Y. *et al.* Risk factors for indigenous Campylobacter jejuni and Campylobacter coli infections in The Netherlands: a case-control study. *Epidemiol Infect.* **138**, 1391-1404 (2010).

9 Doorduyn, Y., Van Den Brandhof, W. E., Van Duynhoven, Y. T., Wannet, W. J. & Van Pelt, W. Risk factors for Salmonella Enteritidis and Typhimurium (DT104 and non-DT104) infections in The Netherlands: predominant roles for raw eggs in Enteritidis and sandboxes in Typhimurium infections. *Epidemiol Infect.* **134**, 617-626 (2006).

10 Ethelberg, S. *et al.* Risk factors for diarrhea among children in an industrialized country. *Epidemiology*. **17**, 24-30 (2006).

11 Clark, S., Berrang-Ford, L. & Lwasa, S. The burden and determinants of self-reported acute gastrointestinal illness in an Indigenous Batwa Pygmy population in southwestern Uganda. *Epidemiol Infect*. **143**, 2287-2298 (2015).

12 Scavia, G., Baldinelli, F., Busani, L. & Caprioli, A. The burden of self-reported acute gastrointestinal illness in Italy: a retrospective survey, 2008-2009. *Epidemiol Infect.* **140**, 1193-1206 (2012).

13 Zhang, J. & Yu, K. F. What's the relative risk? A method of correcting the odds ratio in cohort studies of common outcomes. *Jama.* **280**, 1690-1691 (1998).

14 Efron, B. & Tibshirani, R. Improvements on Cross-Validation: The 632+ Bootstrap Method. *J Am Stat Assoc.* **92**, 548-560 (1997).
